# Maryland State Veterinary Medical Association—Sixth Annual Meeting

**Published:** 1892-03

**Authors:** H. A. Meisner

**Affiliations:** Secretary


H. A. Meisner, V. M. D., *Secretary.*

---

The regular monthly and sixth annual meeting of the Maryland State Veterinary Medical Association was held February 18th, at Tierney's Hotel, 205 N. Calvert street, with the President, Dr. W. H. Martenet, in the chair. The following members responded to roll call : Drs. W. H. Martenet, Wm. Dougherty, Geo. C. Faville, A. W. Clement, T. F. Barron, D. R. Hoffman, and H. A. Meisner. The minutes of the previous meeting were read and approved. Report of Secretary and Treasurer was read and approved. Motion was made and carried that Dr. Dougherty be placed on the Ambulance Committee, which now consists of Drs. A. W. Clement, D. R. Hoffman, and Wm. Dougherty. Dr. Clement withdrew his amendment to Article III, Section 2, and Dr. Geo. Faville made a motion as follows : Graduates of the Veterinary Department of the Baltimore University be not eligible to membership of this society ; carried. Dr. W. H. Martenet then presented an amendment to Article I of the by-laws, which reads : The meetings of this Society shall be held on the third Thursday of each month ; adopted. The Board of Censors reported favorably on the name of Dr. Johnuan, and moved that he be accepted as an active member of this Society ; carried, Dr. Faville was appointed Essayist for next meeting.

The election of officers for the ensuing year resulted in the choice of : President, Dr. W. H. Martenet ; Vice-president, Dr. Geo. C. Faville ; Secretary and Treasurer, Dr. H. A. Meisner ; Board of Censors, Drs. Wm. Dougherty, T. F. Barron, A. W. Clement, D. R. Hoffman.

The meeting then adjourned to retire to the banquet hall, where the members and a number of invited guests did justice to the bountiful repast which awaited them, and so ended the most enjoyable meeting the Society has ever held.

